# Influence of Lennard–Jones Parameters in the Temperature Dependence of Real Gases Diffusion through Nanochannels

**DOI:** 10.3390/nano13091534

**Published:** 2023-05-03

**Authors:** Brais Rodríguez García, Manuel M. Piñeiro, Martín Pérez-Rodríguez

**Affiliations:** CINBIO, Departamento de Física Aplicada, Universidade de Vigo, 36310 Vigo, Spain; brairodriguez@alumnos.uvigo.es

**Keywords:** Lennard–Jones, noble gases, Umbrella Sampling, clathrate, hydroquinone

## Abstract

Umbrella Sampling Molecular Dynamics has been used to determine transition energies for different guest molecules through hydroquinone β-clathrate nanochannels, as well as their temperature trend. This clathrate has been shown to successfully enclathrate different types of small gases with remarkable selectivity, and thus it has been proposed as a potential gas separation and storage medium. Most of these potential guest gases can be successfully modeled as single Lennard–Jones spheres. Then, to obtain a general view of diffusion probabilities for different potential guest molecules, a comparative study for different virtual guest molecules described by different Lennard–Jones parameters has been performed. A regular temperature trend has been obtained for the transition energies for the molecular model characteristic parameter range explored. Finally, to locate the transition energy values of real gases within the space of phases explored, calculations have been repeated for molecular models of different noble gases and H${}_{2}$. The correlation results presented allow a wide interpolation ability for determining the transition energies of potential guest molecules stored or diffusing through the nanochannels of the studied clathrate structure.

## 1. Introduction

A clathrate is a solid structure whose constituent molecules, known as hosts, are arranged in an ordered and periodic lattice such that small guest particles can fit between the voids or cells of the network, thus becoming encapsulated within the matrix. The types of occupancy that can occur within the same cell are divided into single or multiple, depending on whether there are only one or more guests per cavity within the crystalline structure, and these can cross from one cell to another in a slow diffusion process in which a specific energy difference comes into play at each transition. This energy is dependent on the characteristics of the guest molecule. For the same host structure and at similar pressure and temperature conditions, the diffusion potential energy barrier is guest dependent. Understanding the specific diffusion process of each guest within a clathrate can help to extend the increasing applicability of these compounds, as well as being directly related to climate change processes, which are currently very relevant on our planet.

The first record of these compounds dates back to the 19th century, described in laboratory conditions by prominent scientists such as, for instance, Faraday [1]. However, they were first observed in the natural environment in the middle of the 20th century, when the growing energy needs derived from human development forced oil companies to expand their activities to areas with extremely cold climates. It was in these same fossil fuel developments that accumulations of solid matter were reported to clog pipelines, causing flow blockages and even explosions [2,3]. These solid deposits were constituted by methane hydrate, being, by extension of the very definition of clathrate, crystalline compounds of a non-stoichiometric nature whose host matrix is composed entirely of water. Due to the abrupt change in conditions to which the methane hydrate was subjected in its path, the gas contained inside it was suddenly released according to the Joule-Thomson inversion, which describes the variation of the temperature of a system as a function of its volume at constant entropy [2,3,4]. This relationship is expressed by the Joule-Thomson coefficient, which inverts its sign at high pressure values, which means that a gaseous system under thermodynamic conditions such as those described above increases its temperature spontaneously as it expands.

Scientific and industrial interest in such compounds was later stimulated by the discovery of large quantities of methane hydrate in continental permafrost and on the ocean floor, which might appear to be an energy resource to be considered in the first instance [5,6]. However, although the extraction of this gas from ice is feasible, it entails considerable damage to local ecosystems, which is unavoidable because there is a high probability that large amounts of hydrocarbon will be irreversibly released into the environment. One cubic meter of solid hydrate is estimated to be able of releasing about 164 m${}^{3}$ of gaseous methane measured under normal conditions [5,6], a dreaded greenhouse gas with a warming potential 83 times higher than that of CO${}_{2}$ over 20 years, and 30 times over 100 years [7,8,9,10,11].

The consequences of increased atmospheric concentrations of these gases are known to result in a redistribution of the planet’s desert and wetland areas. These changes have been directly linked to an imbalance in the number of pests and diseases affecting flora and fauna alike, including human health [12,13,14]. The already noticeable negative effects on our planet might be aggravated by the decomposition or consumption of methane deposits contained in hydrates. The emission of greenhouse gases into the atmosphere accelerates permafrost decomposition, which leads to the release of more methane in a positive feedback loop. However, despite the doubts about their use as an energy source, the interest generated by hydrates led to the discovery of several valuable applications that have been put into regular practice today, such as wastewater purification and seawater desalination [15].

In parallel to the water-based host structure, organic clathrates offer a wide variety of solid matrices with unique characteristics and distinctive properties. A clear example of a competitive structure from an applied point of view is hydroquinone clathrate (HQ clathrate) [16,17,18], the central objective of this study, which possesses a flexible lattice capable of adapting the cell size to a new guest particle without the need for the system to undergo a phase transition [19,20,21]. Nanostructured materials such as HQ clathrate, due to their peculiar properties, present valuable and important uses, both from scientific and industrial points of view. Among the most common uses are the capture of pollutants, and storage of substances of interest such as fuels, flammable fluids or reagents, that require safe transport [16,17,18,22,23], or absorption of greenhouse gases and solid-liquid separation processes, commonly known as “selective clathration”. The latter is based on the preference of a host structure for a certain substance present in a liquid sample, which is then confined in the solid phase as guest particles [10,18,21,24,25,26,27].

The HQ-based solid and crystalline matrix can act selectively towards moderately complex combinations of gaseous substances, being able to discriminate between them. HQ clathrate has a high affinity towards CO${}_{2}$ molecules, having been empirically observed for the case of a mixture of this gas with methane. This characteristic is considered crucial for its application in atmospheric decarbonization policies, in which it can play a decisive and active role in the removal of atmospheric greenhouse gases [24,27].

In addition, it is worth highlighting the high adaptability of the structure of HQ, which allows the safe storage of substances inside it, some of such importance for current human development as hydrogen [16,17,18,22,23]. Hydrogen, if obtained from renewable sources, is considered a green fuel with a broad future perspective whose use may become standardized in the near future to produce energy for a wide range of fields. The enclathration of hydrogen in an organic network provides a safe storage alternative, avoiding the risks inherent to its explosive nature [18].

Regarding the different phases of HQ solid crystalline phase, three different polymorphs can be distinguished that can occur under normal conditions: α, β, and γ [24,28,29]. The first of them, denoted as α-HQ phase, is conformed by small-size cells able to host low atomic radius guests such as He or Ne and molecules such as CO${}_{2}$ and H${}_{2}$, being this polymorph belonging to the rhombohedral space group R$\bar{3}$. In this first structure, the available space in the structure allows a very limited maximum occupancy, with only a 1:18 guest-to-host ratio, the smallest among all the HQ crystalline phases [24,28,29]. With respect to the β polymorph, three different types of networks are known, usually denoted as type I, II, and III, which share the same host-guest ratio of 1:3 with single full occupancy, with the formation conditions and the guest nature being the determining factors in obtaining them [30,31,32]. The first of them is directly linked to atomic guests such as Ar, Xe, and Kr, the latter belonging to the space group R$\bar{3}$ [24,33]. In turn, the second type belongs to the R3 group and can be observed hosting larger guest molecules such as CH${}_{3}$NC, HCl, and CH${}_{3}$OH. In reference to the type III of the β-HQ, this can be formed by guests similar to acetonitrile, belonging to the P3 group [24,32,33]. In addition, it should be emphasized that the structure of the β-HQ falls into the group of those materials that present ultramicropores, with an estimated diameter of about 0.5 nm. If rapid evaporation in ether or sublimation processes are employed for its synthesis, it is possible to obtain the last of the stable polymorphs of this substance under normal conditions, this being denoted as γ-HQ, a monoclinic crystalline phase of the same [24,34]. It is noteworthy that if the α phase is exposed to high pressures, it is possible to synthesize a fourth phase of HQ corresponding to the δ form [30,31,32]. The highest guest-host ratio of HQ is found in its β phase, being this the one that currently presents the greatest applicability due to the greater availability of space by the guests, easily adapting its dimensions and characteristics to a wide range of guests, allowing a great variety of them to be accommodated in its structure.

The typical diffusion process of any guest within the nanoporous structure of β-HQ consists essentially of two distinct types of movement, differentiated by the scope of their range. The first of these is experienced by the particle within the cavity in which it is contained, defined as a random displacement over a short distance and limited only to the space available in its confinement. On the other hand, the second type of displacement corresponds to the long-term, continuous transition of the guest from one cell to the next through a specific reaction coordinate, usually defined by the typical tubular structure of the β-HQ. It is, therefore, the overall compendium of all hops over time that makes up the effective path of the particle through the crystalline matrix.

However, although all possible guests for a structure have both types of motion within the β-HQ structure, independently of the particle size, intercage diffusion is not always observable using accessible timescales in Molecular Simulation. This can be explained by the energy landscape that the particle experiences along its trajectory in the jump between two cells. In each of the transitions, there is an energy (ΔE) involved that is characteristic of the structure and the type of guest, this being determined by the difference between that corresponding to the configuration of the system in transit. This is characterized by the guest particle being located just between the two cells, this being a situation of considerably higher energy and, therefore, more unstable, and that in which the particle is stable and contained in the equilibrium position inside a single cell. Thus, it is to be expected that if the energy at play during the long-term travel of the guest particle is too high, the transition probability will be considerably low, in what is commonly known as a “rare event”. Consequently, it is unfeasible to obtain a representative and reliable sampling from conventional Molecular Dynamics techniques and, therefore, its study by these methods becomes hardly accessible.

As a feasible solution to this problem, several alternatives have been proposed to correct the poor sampling in the simulated production phase. Among them is a method known as “Umbrella Sampling”, which was chosen for this study due to its compatible properties, thus providing an alternative approach to the study of the problem of interest [35,36,37,38,39]. Using this technique, a specific potential is used as a substitute for the standard Boltzmann weighting, to inhibit the influence exerted by this potential. Umbrella Sampling technique was originally conceived as a way of improving the sampling of complex systems in which two or more regions of space are divided by the action of a specific potential, such as those in which some kind of biological membrane is involved. The technique makes it possible to force the guest to diffuse between adjacent cells by applying a “pull” force [35]. As a result, different configurations are obtained, corresponding to all the intermediate configurations that occur during the transition of the particle through the barrier, which is used as the initial topology for the production phase. Afterward, each of the different configurations obtained in the previous “pull phase” is subjected to a new simulation calculation, keeping the guest particle fixed. By plotting the value for the energy of the system obtained for each of the configurations along the reaction coordinate, it is possible to reconstruct the total energy landscape that the guest particle undergoes during its transit.

In contrast to the great advantages that the Umbrella Sampling technique offers, it requires working on a previously defined trajectory, which implies ignoring possible relevant configurations that may occur outside the previously imposed reaction coordinate, with possible repercussions on the macrostate. In the system formed by the HQ clathrate and several guest particles, there is the possibility of a transit of some of them to one of the so-called “secondary channels”, in which the guest is placed outside the primary channel that originally contained it and, therefore, away from the typical trajectory taken into account by the method. However, the probability associated with this event is very low for situations of multiple occupancy of the network, and is almost completely negligible in single occupancy conditions, as discussed in this paper. Consequently, the Umbrella Sampling technique is a convenient and useful option whose characteristics are remarkably suited to those required, providing the necessary tools to address the objective of this study.

## 2. Computational Methods

Simulations were performed using classical Molecular Dynamics, based on the OPLS-AA (Optimized Potentials for Liquid Simulations-All Atom) forcefield [37,40]. Different models compatible with OPLS-AA forcefield were previously described and tested, both for HQ host [24] and guest molecules [41]. Guest molecules were modeled as neutral spherical particles, therefore, only the Lennard–Jones parameters corresponding to standard van der Waals interaction were used, as expressed in Table 1 for noble gases. In the specific case of H${}_{2}$, to better emulate its real behavior and, at the same time, to study and compare the effect of its distribution in relation to those simpler ones, two centers of mass of 1.008 a.m.u. have been placed on two respective virtual sites located at a distance of 0.032 nm on opposite sides of the Lennard–Jones sphere used, whose initial mass has been redistributed, thus emulating both atomic nuclei. Likewise, two positive charges equivalent to 0.4932 eV have been placed on them and, in addition, a negative charge of −0.9864 eV has been added in the center of both, to emulate the real electron density of the neutral molecule. Following the previous study [41], the methane molecule was also considered to be well as an isolated Lennard–Jones sphere, a good approximation taking into account its shape, symmetry, and electric behavior.

Besides these models representing real gases, theoretical guests were also built for different values of Lennard–Jones parameters, ϵ and σ, named, respectively, as E1 to E6, and S1 to S5, and shown in Table 2, all of them being spherical particles of mass m = 1 a.m.u. These theoretical guests were designed specifically to analyze separately the role of each one of the two characteristic parameters, in the temperature dependence of guest diffusion. The choice of the particular values will be explained in more detail in Section 3. The molecular model for HQ was used as described in our previous works [24].

Velocity re-scaling thermostat [42] was employed for keeping the temperature constant, and the Parrinello–Rahman [43,44] barostat maintained the pressure fixed in NpT simulations. In addition, a semi-isotropic coupling scheme was used, to take into account the different types of mechanical response of the HQ crystal depending on the spatial direction, being *x* equivalent to *y* and different from *z*. Usual periodic boundary conditions and minimum image conventions were considered. Cut-off values for long-range interactions were set to 1.4 nm. The calculation of intermolecular interactions was accelerated using Particle Mesh Ewald (PME) algorithm [45,46], and Lorentz-Berthelot combining rules, in the approach of Good and Hope [47] were used for obtaining the crossed interactions.

**Figure 1:**
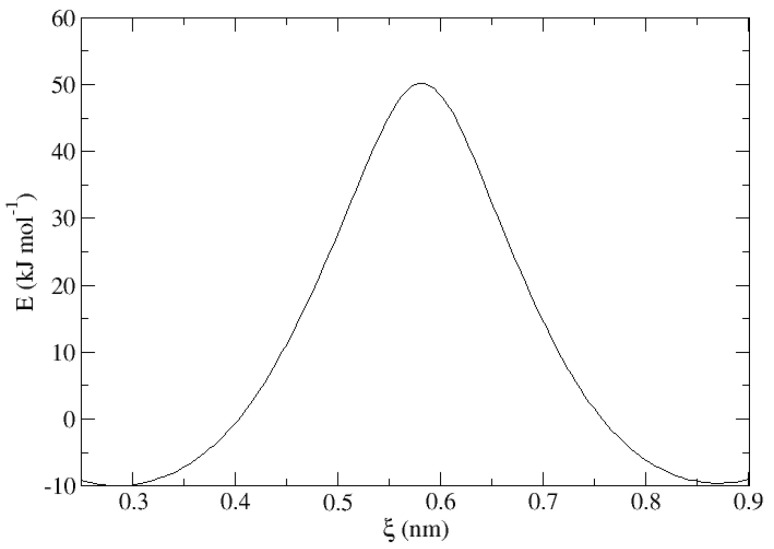
Representation of the energy of the system expressed in kJ·mol${}^{-1}$ along the reaction coordinate ξ expressed in nm in the transition of an Ar atom along the β-HQ crystalline matrix at 380 K and 0.1 MPa.

The Umbrella Sampling (US) method was used as cited to overcome the limitations of the conventional MD simulations in the description of low-probability events. The method WHAM (Weighted Histogram Analysis Method) [48] was used, in the implementation of the Gromacs [49] application, for obtaining the PMF (Potential of the Mean Force) profile of the inter-cell transitions along the HQ nanochannels. The difference between the maximum and minimum of the PMF profile is a very good estimation of the energy transition barrier, ΔG. A clear example of this is shown in Figure 1, corresponding to the graphical representation of the energy of the system expressed in kJ·mol${}^{-1}$ versus the reaction coordinate ξ measured in nm during the transition of an Ar atom through the crystal lattice at 380 K and 0.1 MPa, allowing the calculation of the difference in energy ΔE involved in the process of jumping from one cell to the neighboring one. In a crystalline and periodic structure, with a complete absence of imperfections and taking a minimum degree of occupation of only one particle contained in the matrix, the only possible element of reference within the whole system is the guest itself. Because of this, given a crystal of identical characteristics at all times, the energy involved in the long-range diffusion of the guest through the clathrate channels is dependent only on the position of the guest within the cell and not on which of them is located along a given reaction coordinate, resulting in a symmetrical and periodic energy maximum. This strategy was successfully applied in a recent work [41], where it is described in more detail.

Gromacs [50,51,52], version 2021.5, was the application used to run the simulations, and its companion utilities were the main tools for the analysis of the resulting trajectories, along with some Python and Bash scripting. VMD (Visual Molecular Dynamics) [53,54] with Tachyon [55] ray tracing system was employed in the preparation of molecular images, and XmGrace plotting utility for the 2D graphics. The main simulations were carried out in dual Intel Xeon Ice Lake 8352Y processors—equipped with 64 cores and 256 GiB of volatile memory each—that are part of the CESGA (Supercomputing Center of Galicia) “Finisterrae III” infrastructure.

## 3. Results and Discussion

**Figure 2:**
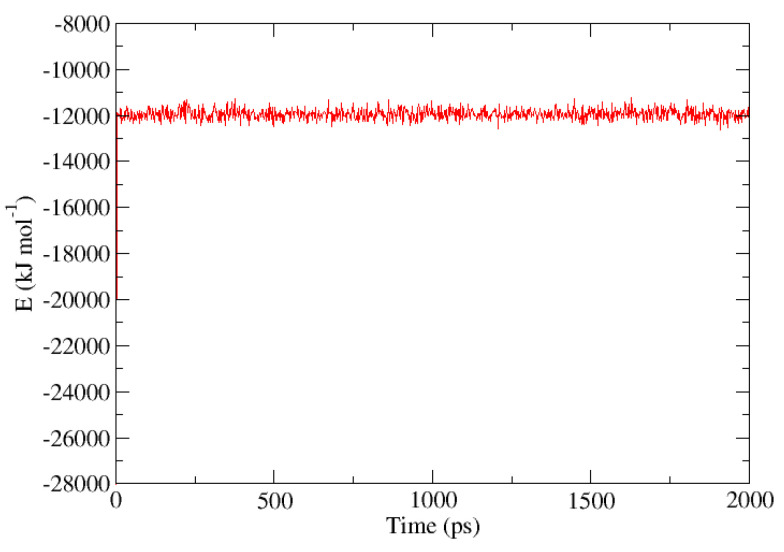
Plot of the energy of the β-HQ system with Ar as a single guest measured in kJ·mol${}^{-1}$ versus the time measured in ps during the equilibration period at 300 K and 0.1 MPa.

**Figure 3:**
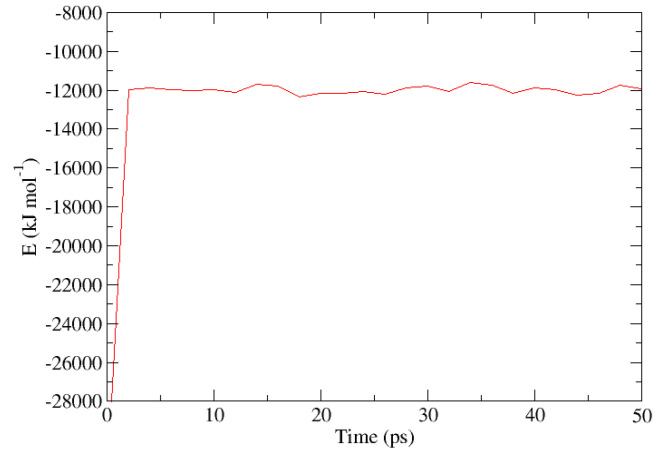
Magnification of the representation corresponding to Figure 2 between 0 and 50 ps simulation in the balancing period for Ar as a guest at 300 K and 0.1 MPa.

**Figure 4:**
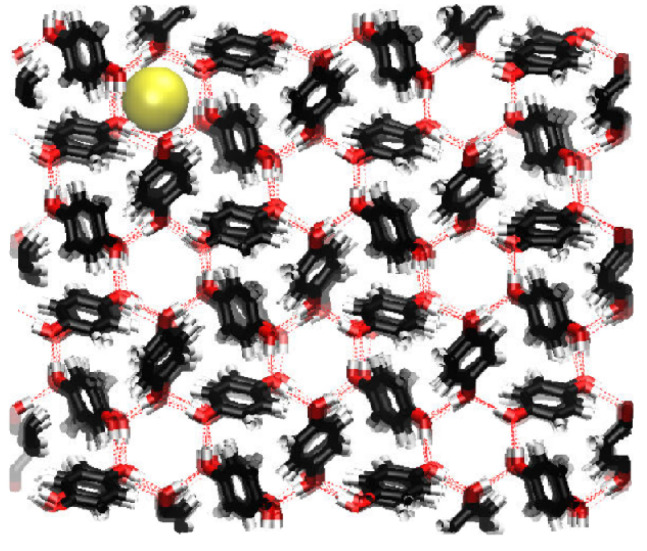
Front view of the β-HQ structure containing Ar as a guest, the latter being represented by the yellow sphere. With reference to the other constituents, C, O, and H atoms are shown as black, red, and white, respectively, while the dashed red lines are representations of hydrogen bonds.

**Figure 5:**
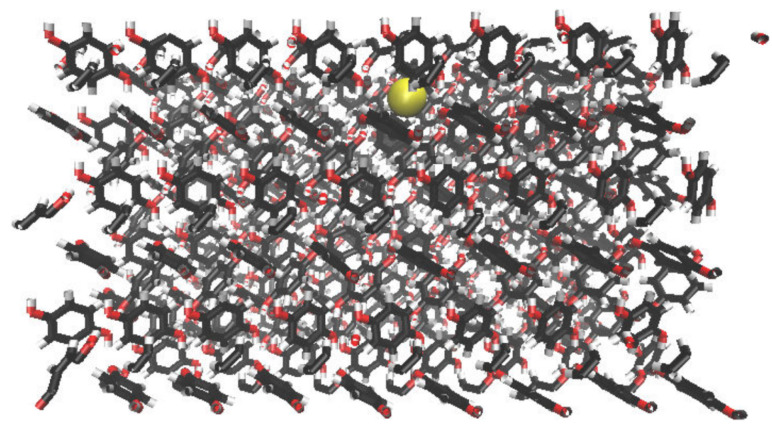
Side view of the β-HQ system with a single Ar atom as guest. The color code is the same than Figure 4.

The initial topology for the β-HQ system has been built from the experimental coordinates of a clathrate containing Xe as a guest obtained by X-ray diffraction [56]. The new guest corresponding to each of the calculations has been placed in the center of a random cell for the subsequent minimization and equilibration process before the pull phase. An example of the representation of the energy of the system expressed in kJ·mol${}^{-1}$ vs. time during the equilibration phase is shown in Figure 2, with Ar as a guest at a temperature of 300 K and at 0.1 MPa, the same pressure at which all the calculations in this study have been carried out. An enlargement of the same between the beginning of the equilibration stage and the first 50 ps of simulation is shown in Figure 3. A sharp increase in energy is observed in the first ps of the simulation, indicating that the system has moved away from its initial configuration. The pseudo-random motion of the HQ molecules constituting the lattice and of the guest itself within its cell entails the loss of the immobile and ideal situation in which the system was after the minimization of the base topology so that the sudden increase and subsequent stabilization of the energy are coherent in the context. Once this equilibrium state is reached, the system is ready to enter the pull phase. Likewise, the image obtained from the positions of each of the atoms contained in the base topology is presented in Figure 4, this being a frontal perspective, and in Figure 5, corresponding to a side view. From the former, it is possible to observe the shape of the cell gates delimited by the hydrogen bonds established between the HQ terminal hydroxyl groups that build up the channels in which the guests are enclathrated.

This procedure has been replicated for each of the calculations for a total of 11 different Lennard–Jones fluids, in a temperature range varying from 220 K to 400 K at 20 K intervals. For the sake of simplicity, these two new types of theoretical guest have been divided into two distinct groups, those named “E” followed by their corresponding number being those where the same value of σ is fixed and ϵ is changed, and, those denoted with “S” are those obtained varying σ but keeping the same value of ϵ in the ensemble. Each of the two different groups proposed contains between five and six members whose names vary in increasing order in relation to the variable parameter of their group, the first number being that guest with the lowest value and the fifth of all of them being reserved for that theoretical particle with the highest assigned value. Both sets of Lennard–Jones fluids have been arranged in such a way as to try to cover a wide spectrum of ϵ and σ values, within which can be found both those intended to define the realistic behavior of small diameter and low interaction capacity guests with the solid matrix, such as He or Ne, as well as those of larger size that exert a greater influence on the nanoporous network such as, for example, Ar. Again, the specific values of σ and ϵ are shown in Table 2.

Given the impossibility of repeating in triplicate each of the calculations due to the large number of simulations involved, an indicative overall sampling standard deviation is obtained for the dataset for a single guest under identical conditions. Table 3 shows those calculations taken in a preliminary way to associate a standard deviation to the proposed Umbrella Sampling method at the points indicated. An attempt has been made to include both a member of the set named “E” and a member of the set named “S”, so that their associated transition energies are considerably different. It is thus possible to study the method uncertainty at different scales within the range in this study. In addition, the calculations have been repeated for two different temperatures, 208 K and 408 K, considering as guest a single H${}_{2}$ atom in the crystalline matrix, since it has the peculiarity of not being defined only as an uncharged Lennard–Jones sphere with a single mass located at its center. Table 3 shows a standard deviation below 0.3 kJ·mol${}^{-1}$ in all cases, so this can be considered an acceptable uncertainty estimation. However, it should be stressed that the uncertainty obtained is merely indicative and can in no case be extrapolated to the overall dataset, as it is a function of the representativeness of the sampling carried out by accumulating weighted histograms from the conditions and the number of simulations carried out to compute this value. However, the uncertainties obtained are considered to be close to each other and comparable in any case, so that the approximate precision of the method can be established around the first decimal place.

**Figure 6:**
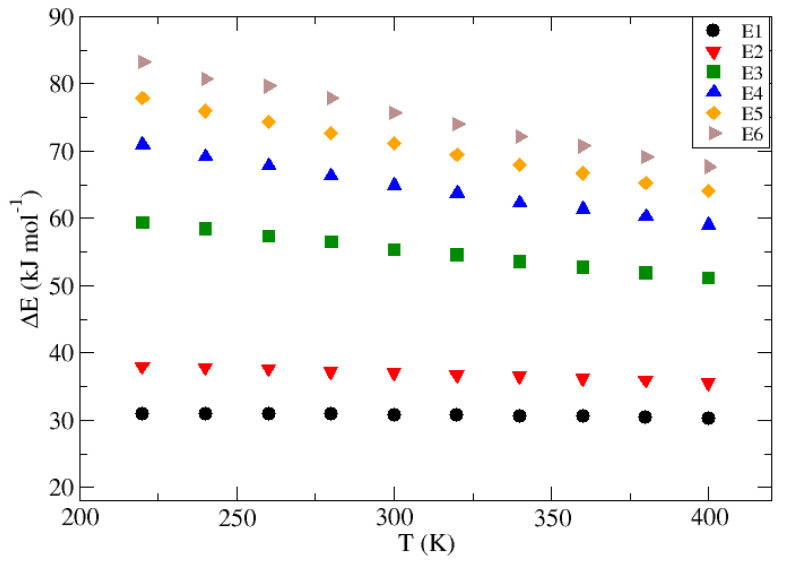
Energy involved in the inter-cell transition (ΔE, kJ·mol${}^{-1}$) for the guests corresponding to group “E” from 220 to 400 K at 20 K intervals.

**Figure 7:**
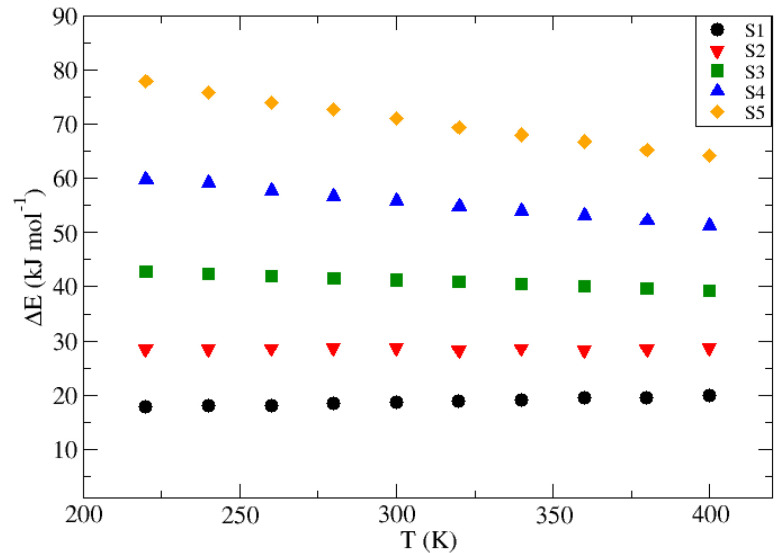
Energy involved in the inter-cell transit (ΔE, kJ·mol${}^{-1}$) for the guests corresponding to group “S” from 220 to 400 K at 20 K intervals.

The temperature trend of the transition energies for the group “E” guests is shown in Figure 6, while those for group “S” guests are represented in Figure 7. Based on these results, it is possible to guess a linear trend between ΔE and temperature for all cases studied. However, both the slope of this correlation and its independent term are guess-dependent, therefore being a function of ϵ and σ LJ parameters. In general terms, higher values of the guest Lennard–Jones coefficients imply a higher independent term, i.e., a higher base energy involved in the transition, but also a higher decay of the latter with increasing temperature, being represented by a steeper slope towards more negative values. This implies that a guest with a larger diameter and higher attraction capacity, such as Ar, has a lower probability of crossing the potential barrier separating two β-HQ lattice cavities, but this will increase with increasing temperature, thus favoring their diffusion. On the other hand, small guests with a low capacity to distort the host structure, such as Ne, will have a higher probability associated with its transition, but this will increase less with increasing temperature or even decrease, as demonstrated by the presence of a positive slope for the particular case of the guest “S1”.

From a physical perspective, the observed effect can be explained through the definition of ΔE, this being the difference in energy between the least stable configurations given in the transit, those in which the guest is at the intermediate point between the center of two contiguous cells, and the most stable, given by those in which the guest particle is contained inside any cell. A general increase in the temperature of the system implies an increase in the energy associated with each of the two configurations described above and, therefore, it can be deduced that the competition between these two increases is what finally determines the slope described by ΔE over the range of temperatures studied. Likewise, taking a single large guest particle with a high attractive capacity, a generalized increase in the kinetic energy of the system leads to a greater destabilization of the most probable configurations with lower energy, thus bringing the two levels closer together and favoring the transition between neighboring cells. In the opposite case, a β-HQ crystalline system containing a guest of very low atomic radius and low attractivity will be hindered in its long-term diffusion with increases in temperature because of a greater destabilization of the configurations corresponding to the particle in transit, thus moving the two states apart and making it less likely that it will occur spontaneously.

The specific results obtained with the Umbrella Sampling technique for the ΔE relative to the diffusion of each of the different Lennard–Jones fluids proposed through the β-HQ porous matrix for each of the temperatures used are shown as a whole in the Table 4 for group “E” and in Table 5 for group “S”. In turn, the slopes (m) and the independent terms (a), as well as their associated errors (Δm and Δa, respectively), and their corresponding correlation coefficients (r${}^{2}$) are presented in Table 6.

The results obtained can be considered consistent, and follow logical and predictable progressions, with fitting uncertainties reasonable and acceptable together with high correlation coefficients. However, the values obtained for the guest “S2” deserve comment, presenting low r${}^{2}$ values, in addition to an error associated with the slope that is higher than its own value. This can be easily associated with the change in sign observed in the slope as the value of σ increases. Since this value is close to zero in this particular case, the usual variations in the ΔE characteristic of the guest transition along the crystalline matrix are overlaid by the uncertainty associated with the method. Far from being an erroneous or discouraging result, it makes sense in the framework discussed, and it is in agreement with the rest of the values obtained, confirming the trend observed in the variation of the dependence of the energy involved in the guest particle diffusion with the temperature at different values of σ. A similar effect is also observed for guest “E1”, but this is less noticeable. In this case, the changes in the slope as the temperature increases or decreases are partly overshadowed by the uncertainty associated with the Umbrella Sampling technique so that even though the value obtained for the slope of the fit is higher than its own error, it is consistently high and has an impact on the respective correlation coefficient.

**Figure 8:**
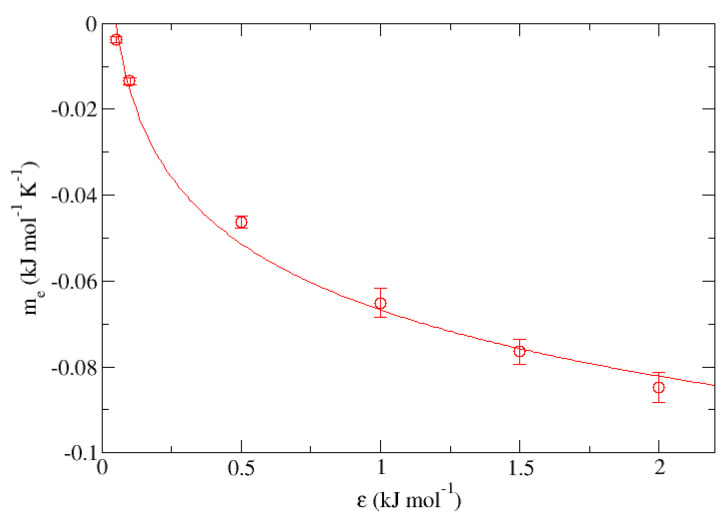
Representation of the slopes obtained from Table 6 for the group “E” (m${}_{e}$) measured in kJ·mol${}^{-1}$·K${}^{-1}$ vs. ϵ expressed in kJ·mol${}^{-1}$.

**Figure 9:**
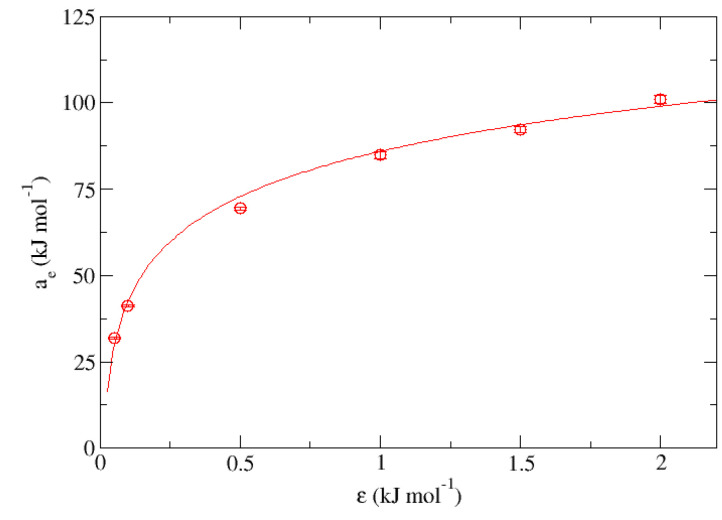
Representation of the independent terms obtained from Table 6 for the group “E” (a${}_{e}$) measured in kJ·mol${}^{-1}$ vs. ϵ expressed in kJ·mol${}^{-1}$.

**Figure 10:**
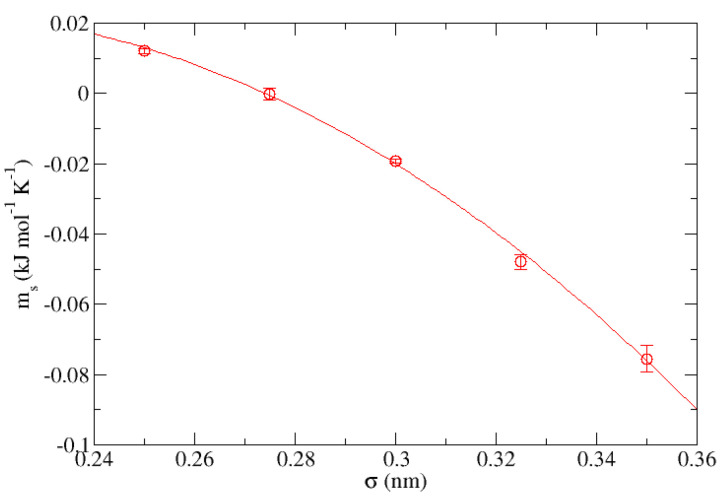
Representation of the slopes obtained from Table 6 for the group “S” (m${}_{s}$) measured in kJ·mol${}^{-1}$·K${}^{-1}$ vs. σ expressed in nm.

**Figure 11:**
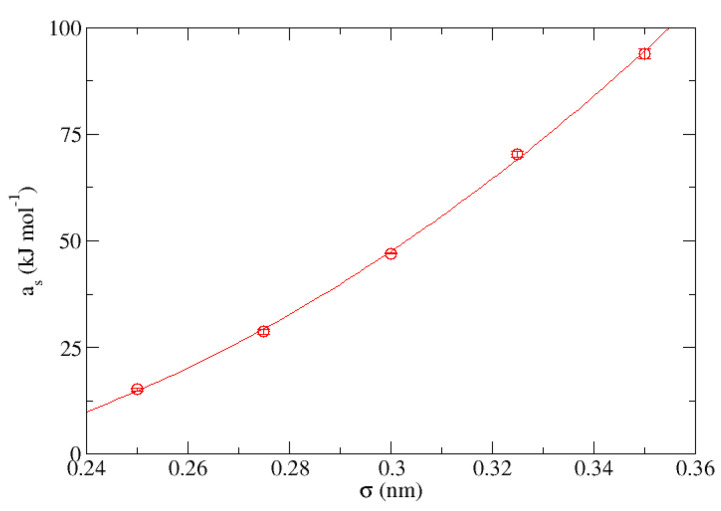
Representation of the independent terms obtained from Table 6 for the group “S” (a${}_{s}$) measured in kJ·mol${}^{-1}$ vs. σ expressed in nm.

Figure 8 and Figure 9 show graphically the representation of “m${}_{e}$” and “a${}_{e}$” obtained from the fits shown in Table 6 for the set “E” vs. ϵ. In the first one, the behavior of the parameter “m${}_{e}$” expressed in kJ·mol${}^{-1}$·K${}^{-1}$ can be described from the equation:

(1) $$m_{e}\left(\epsilon \right)=-0.022\frac{kJ}{mol\cdot K}\cdot ln\left(\frac{\epsilon }{\epsilon_{0}}\right)-0.067\frac{kJ}{mol\cdot K}$$

being $\epsilon_{0}$ an arbitrary parameter used only to keep the dimensionlessness within the natural logarithm with a value of 1 kJ·mol${}^{-1}$, presenting a correlation coefficient r${}^{2}$ = 0.989 for a fixed σ = 0.340. Now turning to the other case, the trend of parameter “a${}_{e}$” corresponding to the independent term of each of the fits made in the set expressed in kJ·mol${}^{-1}$ can be described as:

(2) $$a_{e}\left(\epsilon \right)=18.9\frac{kJ}{mol}\cdot ln\left(\frac{\epsilon }{\epsilon_{0}}\right)+86.0\frac{kJ}{mol}$$

with a correlation coefficient of r${}^{2}$ = 0.993. Comparing both equations, we have that for a given value of σ it is satisfied that ΔE(T,ϵ) = T·m${}_{e}\left(\epsilon \right)+a_{e}\left(\epsilon \right)$. In the same way, the process has been repeated for both parameters for the case of the “S” group at a fixed value of ϵ = 1.000 kJ·mol${}^{-1}$, corresponding in this case to Figure 10 and Figure 11, respectively, being the equations describing the behavior of each of them:

(3) $$m_{s}\left(\sigma \right)=\frac{-0.167\;kJ}{mol\cdot K}+\frac{1.87\;kJ}{mol\cdot K\cdot nm}\cdot \sigma +\frac{-4.6\;kJ}{mol\cdot K\cdot nm^{2}}\cdot \sigma^{2}$$

(4) $$a_{s}\left(\sigma \right)=\frac{65.6\;kJ}{mol}+\frac{-918\;kJ}{mol\cdot nm}\cdot \sigma +\frac{2859\;kJ}{mol\cdot nm^{2}}\cdot \sigma^{2}$$

whose correlation coefficients are r${}^{2}$ = 0.998 and r${}^{2}$ = 0.999, respectively. In the same way, for the aforementioned value of ϵ it is satisfied that ΔE(T,σ) = T·m${}_{s}\left(\sigma \right)+a_{s}\left(\sigma \right)$.

**Figure 12:**
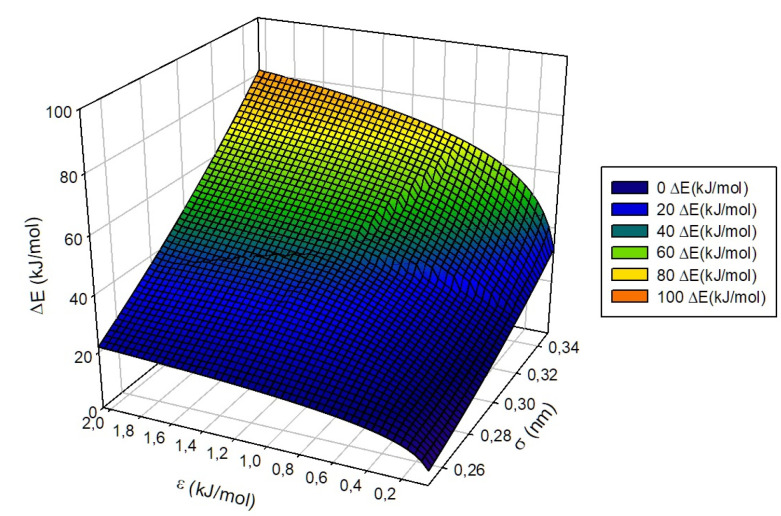
Representation of ΔE surface (kJ·mol${}^{-1}$) trend with σ and ϵ, at T = 300 K.

**Figure 13:**
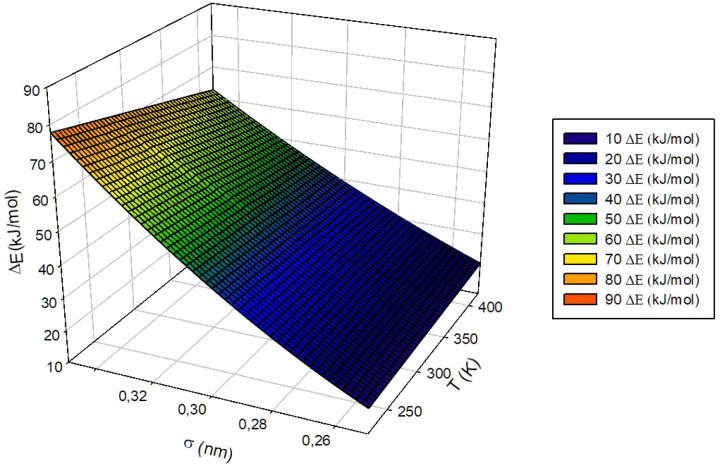
Representation of ΔE surface (kJ·mol${}^{-1}$) trend with σ and temperature, at a fixed σ value of 0.35 nm.

**Figure 14:**
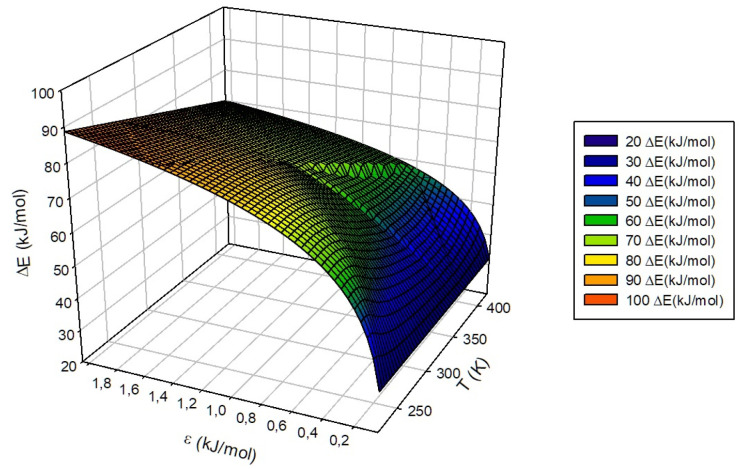
Representation of ΔE surface (kJ·mol${}^{-1}$) trend with ϵ and temperature, expressed in kJ·mol${}^{-1}$ and K, respectively, at a fixed ϵ value of 1.000 kJ·mol${}^{-1}$.

From the combination of the general set of equations obtained from the fits, it is possible to construct a general and predictive expression capable of yielding an interpolated ΔE energy for any guest defined by σ and ϵ parameters at a given temperature within the limits taken into account in this study. Thus, the proposed general fitting function for ΔE within the β-HQ crystalline matrix in the studied ranges is defined as follows:

(5) $$\Delta E\left(T,\sigma ,\epsilon \right)=T\cdot m_{e}\left(\epsilon \right)\cdot \frac{m_{s}\left(\sigma \right)}{m_{e}\left(1\right)}+a_{e}\left(\epsilon \right)\cdot \frac{a_{s}\left(\sigma \right)}{a_{e}\left(1\right)}$$

with m${}_{e}$(1) = −0.066 kJ·mol${}^{-1}K^{-1}$ and a${}_{e}$(1) = 85.1 kJ·mol${}^{-1}$ being constants obtained from the substitution of the variable ϵ in m${}_{e}\left(\epsilon \right)$ and a${}_{e}\left(\epsilon \right)$ by the constant value assigned for the “S” group, i.e., ϵ = 1.0 kJ·mol${}^{-1}$. As a whole, the general behavior of ΔE is defined as a hypersurface whose behavior depends linearly on the temperature and the natural logarithm of ϵ and quadratically on the σ parameter. One of the possible surfaces that can be obtained and isolated from the proposed adjustment is shown in Figure 12, being, in this case, the representation of the behavior of the energy involved in the transit of the guest between adjacent cells defined from the parameters ϵ, measured in kJ·mol${}^{-1}$, and σ, measured in nm, characteristic of the same at a fixed temperature of 300 K. In the same way, it is possible to define and graphically represent the value of the estimated ΔE as a function of temperature and of one of the parameters σ or ϵ, Figure 13 corresponding to the first of both cases mentioned for a ϵ of 1.000 kJ·mol${}^{-1}$ and Figure 14 to the second of them for σ = 0.35 nm.

**Figure 15:**
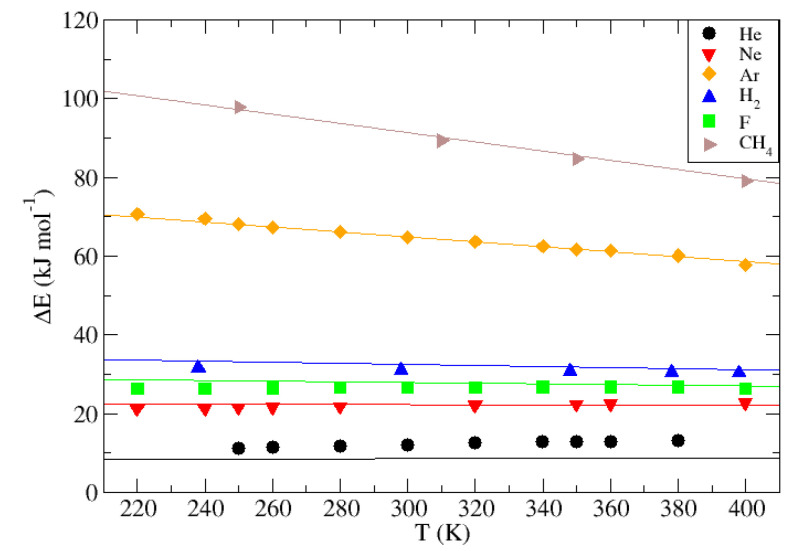
ΔE (kJ·mol${}^{-1}$) of the different representative guests of real molecules at different temperatures compared to their extrapolated value from the correlation (solid lines).

To test the fit from the two different sets of proposed Lennard–Jones theoretical fluids, a similar procedure has been carried out for different guests whose characteristic Lennard–Jones values, collected and related to their respective guest in the aforementioned Table 1, simulate the behavior of real atoms or molecules through the β-HQ matrix. Different point values corresponding to the energy at play in the guest transition along the crystalline structure have been taken at different temperature intervals between 220 K and 400 K. The results obtained are shown as points on the graphical representation in Figure 15, while solid lines correspond to those calculated from the correlation obtained applied to the proposed temperature range, taking as values of σ and ϵ those corresponding to each of the different guests.

The results obtained show that the larger errors are associated with the configurations at low temperatures. At these conditions the energy levels of the key configurations of the system at the transition between cells are farther apart and, therefore, the sampling must overcome a higher potential barrier, making it more difficult to obtain representative energy sampling. This effect can be easily observed for hosts such as Ne, F, or H${}_{2}$.

An underestimation is also observed of the correlated He values, compared to those obtained directly from the Umbrella Sampling technique. This may be due to poor sampling occasionally observed in particles whose transit energy is consistently low, causing their hopping between neighboring cells to become spontaneous and observable with conventional Molecular Simulation times. Consequently, the particle traverses those areas crucial for the proper description of the energy landscape along the reaction coordinate ξ too fast. Nevertheless, a direct execution of the method limiting the maximum displacement of the particle per unit of time does not guarantee in any case that the particle transits from one cell to another in the required period and, if it does, it is difficult to obtain a sampling good enough to be comparable with those guests with higher ΔE associated. Because of this, it is possible that the fit corresponding to low values of ΔE is subject to comparatively larger error, corresponding to the nonlinear and hence more sensitive areas of the logarithmic fits with ϵ parameter. However, in absolute terms, the largest error committed is 4.5 kJ·mol${}^{-1}$ for, again, the case of He, and not exceeding 2 kJ·mol${}^{-1}$ in any other case. Therefore, the results extracted from the fit are considered to adequately agree with those obtained from the realistic guest simulation.

Considering the specific case of H${}_{2}$, the only guest that has not been represented by a single neutral Lennard–Jones sphere, it is observed that the error committed by the correlation is comparable with the rest and, therefore, it is considered that the results extracted from this are acceptable and applicable to this model in the proposed range, thus providing a more accurate description of a molecule with partial charges and a realistic mass distribution. This result is of great importance, as it allows the described equation to be used to define the behavior within the crystalline matrix of β-HQ of a molecule of great interest from an applied point of view at a scientific and industrial level.

For the particular ΔE value obtained for Ar, it was possible to compare the result obtained from the correlation with the data obtained by Woo et al. [35]. Experimentally, the result obtained is 79.1 kJ·mol${}^{-1}$, while using Molecular Simulation the value of the energy involved in the diffusion is between 60.0 and 65.0 kJ·mol${}^{-1}$ for a range of temperature conditions between 269 K and 390 K. The extrapolated result of the fit for Ar at a temperature of 300 K is 64.9 kJ·mol${}^{-1}$ and 63.6 kJ·mol${}^{-1}$ at 320 K, while that calculated directly in this report by Molecular Simulation methods at 300 K is 64.8 and 63.6 at 320 K. The results obtained in this study are within the described range obtained by similar methods, and are very close to those described experimentally, supporting the calculation scheme used in this case.

## 4. Conclusions

The variation in the transition energy between HQ clathrate cells at different temperatures of a group of guest substances made up of Lennard–Jones fluids has been computed using the Umbrella Sampling technique. To analyze the influence of the guest characteristic Lennard–Jones parameters, two sets of theoretical guest molecules were explored: “E”, those whose progression keeps the value of σ fixed and allows ϵ to vary in an increasing way from “E1” to “E6”, and “S”, in which, on the contrary, ϵ remains constant for all “S” while σ varies from “S1” to “S5”. Through the application of a pull force, this technique allows the sampling and consequent study of all those configurations which, due to their instability, are not easily accessible by typical Molecular Simulation methods, being especially useful in those systems in which a potential barrier separates two well-differentiated areas of space. The energy involved in the transition has been calculated for each of the 11 proposed Lennard–Jones fluids at temperature intervals of 20 K within the range 220 to 400 K. A linear temperature trend has been observed relating the results obtained for this transition energy in the range studied and, subsequently, the slopes (m${}_{e}$ and m${}_{s}$) and the respective independent terms (a${}_{e}$ and a${}_{s}$) have been plotted against Lennard–Jones parameters within each of the two sets of fluids proposed, thus observing that they follow a quadratic relationship against the σ parameter and a linear one against the natural logarithm of ϵ. Using the equations obtained from each of the correlations made, it was possible to obtain an expression corresponding to a hypersurface that summarizes all the trends observed as a function of temperature and the guest Lennard–Jones, as described in Equation (5).

A similar process has been repeated at different temperatures within the proposed range for different realistic guest particles able to emulate the real behavior of their respective fluid from Molecular Simulation methods, these being: He, Ne, F, H${}_{2}$, Ar, and CH${}_{4}$. All of them have been defined as uncharged Lennard–Jones spheres with a single mass located at their center, except in the case of H${}_{2}$, which has been treated more realistically. Its behavior has been defined as that of a Lennard–Jones sphere with a distribution in which two centers of mass of 1.008 a.m.u. with a charge equivalent to 0.4932 eV are both in line at a distance of 0.032 nm from its center in which a charge of −0.9864 eV has been placed, resulting in a neutral particle. The results obtained have been compared with those extrapolated from the fit, considering these to be adequate and sensitive and generally observing a lower precision proportional to a low value of temperature and ΔE associated with the guest particle, but being, regardless of this, appropriate and of a similar order. In the case of H${}_{2}$, the range of concordance is similar, so it has been considered that the fit, despite having been proposed only for models defined by single Lennard–Jones spheres, is able of yielding reliable results consistent with those obtained by a more complex and realistic model for this particular molecule and within the limits studied. Likewise, it has been possible to compare the value obtained for Ar with those existing in the literature, having been obtained both by Molecular Simulation and experimentally, observing an adequate coincidence.

Thus, the method applied in this work can be used in a broader context to estimate different gas diffusion properties inside solid materials. As shown by the results presented here, the Umbrella Sampling theoretical approach can describe with accuracy even low-probability transition phenomena for this type of system, revealing itself as a valuable tool to explore processes, such as solubility or permeability, just to cite a couple of representative and interesting examples.

## Figures and Tables

**Table 1:** OPLS atom type used and force field parameters [37], mass, σ and ϵ, for the guest molecules considered in the simulations.

Guest	Type [37]	Mass/a.m.u.	σ/nm	ϵ/kJ·mol${}^{-1}$
He	opls_130	4.003	0.256	0.08368
Ne	opls_129	20.180	0.278	0.2887
Ar	opls_097	39.948	0.340	0.9786
F	opls_965	18.998	0.295	0.2218
CH${}_{4}$	opls_066	16.043	0.373	0.1230
H	opls_013	1.008	0.304	0.2852

**Table 2:** Lennard–Jones parameters of each of the theoretical proposed guests, where σ is expressed in nm and ϵ in kJ·mol${}^{-1}$.

L-J Parameters/Guest	E1	E2	E3	E4	E5	E6
**σ**/nm	0.340	0.340	0.340	0.340	0.340	0.340
**ϵ**/kJ·mol${}^{-1}$	0.05000	0.1000	0.5000	1.000	1.500	2.000
**L-J parameters/Guest**	**S1**	**S2**	**S3**	**S4**	**S5**	-
**σ**/nm	0.250	0.275	0.300	0.325	0.350	-
**ϵ**/kJ·mol${}^{-1}$	1.000	1.000	1.000	1.000	1.000	-

**Table 3:** In which three measurements are given for the energy involved in the transit of various guests between two cells together with their respective sample standard deviation (S.S.D.) expressed in kJ·mol${}^{-1}$ according to the indicated temperature.

Guest	T (K)	Measurement Number	ΔE/(kJ·mol${}^{-1}$)	S.S.D./(kJ·mol${}^{-1}$)
		1	59.5	
E4	400	2	59.0	0.2
		3	59.3	
		1	19.58	
S1	360	2	19.60	0.01
		3	19.58	
		1	31.57	
H${}_{2}$	208	2	31.41	0.09
		3	31.43	
		1	30.11	
H${}_{2}$	408	2	30.01	0.08
		3	30.16	

**Table 4:** ΔE, (kJ·mol${}^{-1}$) for the proposed guests belonging to the group “E” vs. temperature.

T	ΔE(E1)	ΔE(E2)	ΔE(E3)	ΔE(E4)	ΔE(E5)	ΔE(E6)
(K)	(kJ·mol${}^{-1}$)	(kJ·mol${}^{-1}$)	(kJ·mol${}^{-1}$)	(kJ·mol${}^{-1}$)	(kJ·mol${}^{-1}$)	(kJ·mol${}^{-1}$)
220	31.0	38.0	59.4	70.9	77.8	83.2
240	31.0	37.8	58.4	69.2	75.9	80.8
260	31.0	37.6	57.3	67.8	74.3	79.6
280	30.9	37.2	56.5	66.3	72.6	77.9
300	30.8	37.1	55.3	64.8	71.1	75.7
320	30.7	36.8	54.6	63.7	69.5	74.0
340	30.6	36.6	53.6	62.3	68.0	72.2
360	30.6	36.2	52.8	61.4	66.7	70.7
380	30.4	35.8	51.9	60.3	65.3	69.1
400	30.3	35.6	51.1	59.3	64.0	67.7

**Table 5:** ΔE, (kJ·mol${}^{-1}$) for the proposed guests belonging to the group “S” vs. temperature.

T	ΔE(S1)	ΔE(S2)	ΔE(S3)	ΔE(S4)	ΔE(S5)
(K)	(kJ·mol${}^{-1}$)	(kJ·mol${}^{-1}$)	(kJ·mol${}^{-1}$)	(kJ·mol${}^{-1}$)	(kJ·mol${}^{-1}$)
220	17.8	28.5	42.9	59.8	77.9
240	18.0	28.6	42.4	59.2	75.8
260	18.1	28.6	41.9	57.7	73.9
280	18.5	28.7	41.6	56.7	72.8
300	18.7	28.7	41.3	55.9	71.0
320	19.1	28.4	40.9	54.7	69.4
340	19.2	28.7	40.5	53.9	68.0
360	19.6	28.3	40.1	53.1	66.8
380	19.6	28.5	39.7	52.2	65.3
400	19.9	28.7	39.3	51.3	64.1

**Table 6:** Linear coefficients of ΔE vs. T correlation (ΔE = m T + a).

Guest	m/kJ·mol${}^{-1}$ ·K${}^{-1}$	Δm/kJ·mol${}^{-1}$·K${}^{-1}$	a/kJ·mol${}^{-1}$	Δa/kJ·mol${}^{-1}$	r${}^{2}$
E1	−3.9·10${}^{-3}$	±7·10${}^{-4}$	31.8	±0.2	0.94
E2	−1.38·10${}^{-2}$	±8·10${}^{-4}$	41.1	±0.3	0.993
E3	−4.6·10${}^{-2}$	±1·10${}^{-3}$	69.4	±0.5	0.998
E4	−6.5·10${}^{-2}$	±3·10${}^{-3}$	85	±1	0.995
E5	−7.6·10${}^{-2}$	±3·10${}^{-3}$	94.2	±0.9	0.997
E6	−8.6·10${}^{-2}$	±3·10${}^{-3}$	102	±1	0.997
S1	1.20·10${}^{-2}$	±8·10${}^{-4}$	15.1	±0.3	0.991
S2	−0.3·10${}^{-3}$	±2·10${}^{-3}$	28.6	±0.5	0.018
S3	−1.93·10${}^{-2}$	±6·10${}^{-4}$	47.0	±0.2	0.998
S4	−4.8·10${}^{-2}$	±2·10${}^{-3}$	70.3	±0.7	0.996
S5	−7.5·10${}^{-2}$	±4·10${}^{-3}$	94	±1	0.995

## Data Availability

Not applicable.
